# An MRI-Based Clinical-Perfusion Model Predicts Pathological Subtypes of Prevascular Mediastinal Tumors

**DOI:** 10.3390/diagnostics12040889

**Published:** 2022-04-02

**Authors:** Chia-Ying Lin, Yi-Ting Yen, Li-Ting Huang, Tsai-Yun Chen, Yi-Sheng Liu, Shih-Yao Tang, Wei-Li Huang, Ying-Yuan Chen, Chao-Han Lai, Yu-Hua Dean Fang, Chao-Chun Chang, Yau-Lin Tseng

**Affiliations:** 1 Department of Medical Imaging, National Cheng Kung University Hospital, College of Medicine, National Cheng Kung University, Tainan 704, Taiwan; gracelinchiaying@msn.com (C.-Y.L.); leating2001@yahoo.com.tw (L.-T.H.); taicheng100704@yahoo.com.tw (Y.-S.L.); 2 Division of Thoracic Surgery, Department of Surgery, National Cheng Kung University Hospital, College of Medicine, National Cheng Kung University, Tainan 704, Taiwan; b85401067@gmail.com (Y.-T.Y.); enozic@hotmail.com (W.-L.H.); ethaneyyc@gmail.com (Y.-Y.C.); tsengyl@mail.ncku.edu.tw (Y.-L.T.); 3 Division of Trauma and Acute Care Surgery, Department of Surgery, National Cheng Kung University Hospital, College of Medicine, National Cheng Kung University, Tainan 704, Taiwan; 4 Division of Hematology and Oncology, Department of Internal Medicine, National Cheng Kung University Hospital, College of Medicine, National Cheng Kung University, Tainan 704, Taiwan; teresa@mail.ncku.edu.tw; 5 Department of Biomedical Engineering, National Cheng Kung University, Tainan 704, Taiwan; sytang@fanglab.bme.ncku.edu.tw; 6 Department of Surgery, National Cheng Kung University Hospital, College of Medicine, National Cheng Kung University, Tainan 704, Taiwan; d303878@mail.hosp.ncku.edu.tw; 7 Department of Radiology, University of Alabama at Birmingham, Birmingham, AL 35294, USA

**Keywords:** differential diagnosis, dynamic contrast-enhanced MRI, perfusion parameters, prevascular mediastinal tumor, machine learning

## Abstract

This study aimed to build machine learning prediction models for predicting pathological subtypes of prevascular mediastinal tumors (PMTs). The candidate predictors were clinical variables and dynamic contrast–enhanced MRI (DCE-MRI)–derived perfusion parameters. The clinical data and preoperative DCE–MRI images of 62 PMT patients, including 17 patients with lymphoma, 31 with thymoma, and 14 with thymic carcinoma, were retrospectively analyzed. Six perfusion parameters were calculated as candidate predictors. Univariate receiver-operating-characteristic curve analysis was performed to evaluate the performance of the prediction models. A predictive model was built based on multi-class classification, which detected lymphoma, thymoma, and thymic carcinoma with sensitivity of 52.9%, 74.2%, and 92.8%, respectively. In addition, two predictive models were built based on binary classification for distinguishing Hodgkin from non-Hodgkin lymphoma and for distinguishing invasive from noninvasive thymoma, with sensitivity of 75% and 71.4%, respectively. In addition to two perfusion parameters (efflux rate constant from tissue extravascular extracellular space into the blood plasma, and extravascular extracellular space volume per unit volume of tissue), age and tumor volume were also essential parameters for predicting PMT subtypes. In conclusion, our machine learning–based predictive model, constructed with clinical data and perfusion parameters, may represent a useful tool for differential diagnosis of PMT subtypes.

## 1. Introduction

Prevascular mediastinal tumors (PMTs), previously known as anterior mediastinal tumors [1], pose diagnostic challenges for clinicians because they are relatively uncommon, making up less than 1% of tumors [2], and because they include a wide variety of entities, including thymomas, benign cyst, lymphomas, and thymic carcinomas [3,4]. Patients with PMT may present chest pain, dyspnea, cough, fever, and/or chills, but many patients are asymptomatic [5]. Some types of PMTs, such as thymic or pericardial cyst, mature teratoma, thymolipoma, thymic hyperplasia, and intrathoracic goiter, have distinguishable radiological features that can be identified at CT and MR imaging [3,6,7,8]. But, when imaging findings were not definitive, biopsy is often required for histological confirmation before initiation of treatment. However, the workup for distinguishing lymphoma from thymoma is a clinical conundrum. Guidelines indicate that lymphoma should be treated medically instead of through surgical resection [9,10,11], and resectable thymic epithelial tumors (TETs) should be surgically resected to avoid tumor seeding from an encapsulated thymoma during the biopsy procedure [6,7]. These are two major risks for managing patients with presumed resectable PMTs. Therefore, a reliable noninvasive approach for differentiating lymphomas from thymic malignancies is in urgent need.

Thymic lymphomas are blood cancers originating from the thymus gland and containing Hodgkin’s lymphomas and non-Hodgkin’s lymphomas [8]. Thymomas and thymic carcinomas are classified as TETs because both of them arise from epithelial cells on the outer surface of the thymus [3]. Thymomas often grow slowly and rarely spread beyond the thymus, but thymic carcinoma is more aggressive with worse prognosis [12,13]. Thus, distinct PMT subtypes require different therapeutic strategies [13,14]. Even when performed by experienced chest radiologists, the diagnostic accuracy of CT for differentiating PMTs was 61%, while the combination of CT and MRI improved the diagnostic accuracy up to 67% [15]. Although CT remains the workhorse of diagnostic imaging for PMTs, multiple lines of evidence indicate that MRI has better soft tissue contrast and that advanced MR techniques, such as chemical shift MRI and diffusion-weighted MRI, can improve the accuracy of differential diagnosis of PMTs [3,16].

Notably, the usefulness of dynamic contrast–enhanced MRI (DCE-MRI) in the diagnosis and prognosis of PMTs was suggested [17]. Quantitative perfusion parameters calculated from DCE-MRI are used to assess vascularity characteristics, capillary permeability, and changes in vascular structure and function [18,19]. The commonly used perfusion parameters included the efflux rate constant from blood plasma into the tissue extravascular extracellular space (EES) (K_trans_), EES volume per unit volume of tissue (V_e_), blood plasma volume per unit volume of tissue (V_p_), efflux rate constant from tissue EES into the blood plasma (K_ep_), and time to the peak of the concentration curve (TTP) [20]. Perfusion parameters have been widely utilized for a variety of clinical applications, such as cancer diagnosis and prognosis [21,22], distinguishing melanoma from lung cancer brain metastases [23], and monitoring tumor progression [19]. The potential of DCE-MRI–derived perfusion parameters for differentiating between thymic lymphoma and thymic carcinoma has been recently demonstrated [24], but the differential diagnostic value of perfusion parameters in multiple PMT subtypes has been rarely investigated.

Due to advances in machine learning, the feasibility of imaging modalities in differential diagnosis of a wide range of disorders has been improved, including Parkinson’s disease [25], lung cancer [26], breast lesions [27], rheumatic and musculoskeletal diseases [28], seizures [29], and meningitis [30]. Moreover, machine learning predictive models can reduce the burden of human effort and costs. Machine learning algorithms can be classified as black-box or white-box [31]. Compared to black-box models built by support vector machine (SVM), neural network, or random forest (RF), white-box decision tree models are self-explanatory, interpretable, and visualizable [31,32].

The aim of this retrospective pilot study was to build DCE-MRI–derived perfusion parameter–based decision tree models for differentiating PMT subtypes. Furthermore, the potential role of age and tumor diameter in differential diagnosis of PMT subtypes has been suggested [33,34], so age at MRI scan and tumor size characteristics were also included as candidate predictors for modelling.

## 2. Materials and Methods

### 2.1. Study Participants

The research protocol was reviewed and approved by the Institutional Review Board of National Cheng Kung University Hospital (B-ER-109-514), and informed consent was waived due to the retrospective nature of this study. Consecutive patients with PMTs and undergoing chest DCE-MRI prior to treatment at National Cheng Kung University Hospital from March 2018 to August 2020 were included. Patients with germ cell tumor, metastatic tumor, thymic cyst, thymic hyperplasia, or ectopic thyroid were excluded, because these PMTs could be diagnosed straightforwardly via distinguishable imaging features, clinical history, and biochemical exams. In addition, (i) patients with benign lesion, (ii) patients with a mass less than 2 cm in diameter due to inherent motion artifact in chest MR, and (iii) patients who could not tolerate the contrast agent due to renal insufficiency were also excluded.

### 2.2. Study Variables

From medical records, patients’ sex, age, cancer treatment, and pathological subtype of PMT subtype were recorded. The subtypes of PMT were determined based on pathologic examination of surgical excision biopsies. Ten parameters, including age; six perfusion variables; and three dimensional variables of the tumor were used to build predictive models for differentiating PMT subtypes via a machine-learning approach.

### 2.3. Chest DCE-MRI Protocol

All included patients underwent chest DCE-MRI using the 3 Tesla system with a 16-channel dStream anterior coil and a 12-channel dStream posterior coil (Ingenia, Philips Healthcare, Best, The Netherlands). The routine MRI sequences included axial multi-echo Dixon; electrocardiogram-gated double inversion recovery T2-weighted sequence; DW imaging at b values of 0, 400, and 800 s/mm^2^; and fat-suppressed T1-weighted imaging.

A DCE sequence was performed using 3D T1-fast field echo (repetition time: 4 msec; echo time: 2 msec; number of excitations: 1; slice thickness: 5 mm with no gap; field of view: 350 mm × 257 mm; bandwidth: 717.4 Hz; acquisition matrix: 176 mm × 128 mm; flip angle: 5° and 15°; dynamic scan time: 2.5 s/image; acquisition duration: 3 min 24 s). DCE T1-weighted images were acquired after bolus administration of gadolinium (0.1 mmol/kg; Gadovist, Bayer Healthcare, Leverkusen, Germany), at a rate of 2 cc/s, followed by 25 mL saline chase. Axial and sagittal T1-weighted contrast enhanced MRI scans were acquired after DCE perfusion MRI. Subtraction imaging was then processed to detect subtle areas of enhancement.

### 2.4. DCE-MRI Image Analysis

The region of interest (ROI) on each DCE-MRI image was traced by the same radiologist using Matlab^®^ software (MathWorks, Natick, MA, USA). Arterial input function was manually selected by defining an ROI at the descending thoracic aorta at right pulmonary artery level on axial images. For each patient, his/her DCE-MRI images were then superimposed to create 3D volume of interest for calculating perfusion parameters. While circling the ROI, other MRI images (T2W, T1W pre- and post-contrast images) were also considered in order to avoid including necrotic tissues. The intensity of volume of interest was converted into the concentration of gadolinium using Bloch’s equation, and perfusion parameters were then calculated via the Matlab function nonlinear least squares curve fitting in an extended Tofts and Kermode model, as previously described [35].

Six perfusion parameters, including K_trans_, K_ep_, V_e_, V_p_, TTP, and maximum concentration in the volume of interest, were calculated from DCE-MRI images. In addition, tumor volume, surface area of the tumor, and maximum diameter of the tumor were also calculated from DCE-MRI images.

### 2.5. Statistical Analysis

Patients’ age is expressed as mean ± standard deviations with range (min. to max.). The other demographic and clinical characteristics are presented as n (%). Ten parameters used for model construction between two subtypes are expressed as median with inter-quartiles, and differences between two pathological subtype groups were examined using the Mann-Whitney U test. After univariate receiver operating characteristic (ROC) curve analysis, the value of area under the curve (AUC) with 95% confidence intervals was used to measure the ability of the corresponding classification to distinguish between PMT subtypes.

The classification and regression tree (CART), a predictive algorithm, was used to construct decision tree models for differentiating between PMT subtypes. The normalized importance for each independent variable was calculated to rank its importance in predicting PMT subtypes. The hyper-parameters of three decision tree models are summarized in Supplementary Table S1. All 10 parameters are input; tree and confusion matrix are output. The sensitivity, specificity, and total accuracy rate of each predictive model were calculated.

Finally, multivariate ROC curve analysis was performed based on the decision tree model to evaluate the abilities of various predictive models. All statistical assessments were two-tailed and considered significant as *p* < 0.05. For multiple comparisons, the false discovery rate was controlled. Statistical analysis was performed using IBM SPSS statistical software version 22 for Windows (IBM Corp., Armonk, NY, USA).

## 3. Results

**Figure 1:**
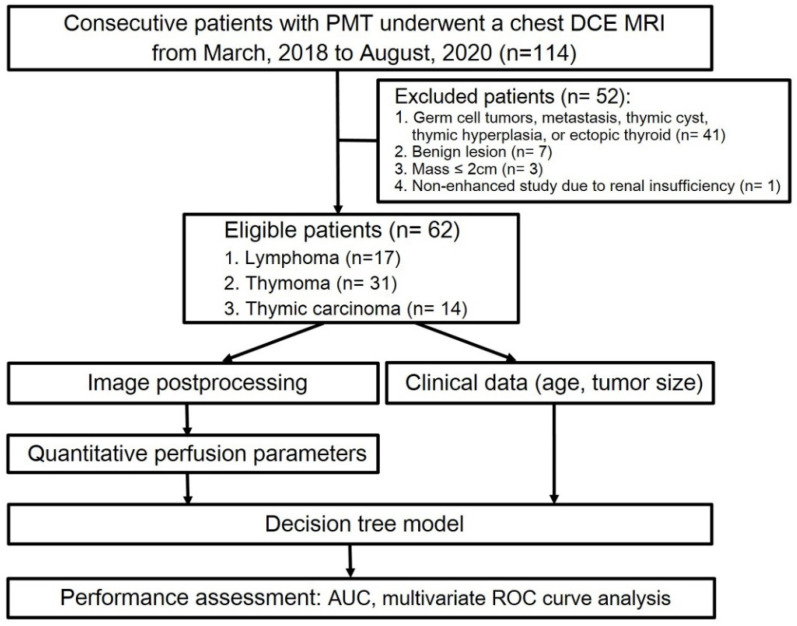
Flow diagram of patient selection and experimental procedure. Abbreviations: PMT = prevascular mediastinal tumor; TET = thymic epithelial tumor.

In this retrospective study, 114 patients who underwent chest MRI due to suspected PMT were initially selected. Among them, 41 patients who had germ cell tumor, metastatic tumor, thymic cyst, thymic hyperplasia, or ectopic thyroid; 7 patients who received chemotherapy prior to the chest MRI scan; 3 patients with a mass less than 2 cm in diameter; and 1 patient who could not receive contrast agent due to renal insufficiency were excluded. As a result, 62 patients diagnosed with PMT, consisting of 28 males and 34 females, were included in the final analysis (Figure 1).

### 3.1. The Demographic and Clinical Characteristics

The mean age of the 62 eligible patients was 52.3 years, ranging from 22 to 82 years (Table 1). Of them, 34 patients underwent surgery, and 28 patients with unresectable PMT received chemotherapy. According to pathological examination of the biopsies, 17 out of 62 patients were diagnosed with lymphoma and 45 were diagnosed with TET. Among 17 patients with lymphoma, 6 patients had Hodgkin lymphoma and 11 had non-Hodgkin lymphoma. Of 45 TET patients, 31 patients had thymoma and 14 had thymic carcinoma. Moreover, 31 cases of thymoma consisted to 25 noninvasive cases (Masaoka stages 1 & 2) and 6 invasive cases (Masaoka stages 3 & 4) (Table 1).

### 3.2. Comparison of Parameters Used for Model Construction between Patients with Different PMT Subtypes

Age, MRI-derived perfusion parameters, and tumor dimension data were compared in patients with different PMT subtypes in Table 2. Compared to patients with TET, patients with lymphoma were significantly younger and had significantly larger tumor volume and surface and longer maximum diameter (all *p* value < 0.05, Table 2). On the other hand, patients with thymoma had significantly lower K_trans_, V_e_, and TTP, but significantly higher K_ep_, than patients with thymic carcinoma (all *p* value < 0.05, Table 2).

### 3.3. Univariate ROC Curve Analysis

Univariate ROC curve analysis revealed that the top three parameters for distinguishing TET from lymphoma were age, maximum diameter, and surface area (AUC = 0.832, 0.780, and 0.684, respectively) (Supplementary Table S2). V_e_, TTP, and K_ep_ were the top three parameters for distinguishing thymic carcinoma from thymoma (AUC = 0.802, 0.779, and 0.765, respectively). Tumor volume, surface area, and age were the top three parameters for distinguishing Hodgkin from non-Hodgkin lymphoma (AUC = 0.848, 0.833, and 0.811, respectively). Finally, maximum diameter, TTP, and surface area were the top three parameters for distinguishing invasive from noninvasive thymoma (AUC = 0.820, 0.813, and 0.800, respectively) (Supplementary Table S2).

### 3.4. Analysis of Variable Importance

**Figure 2:**
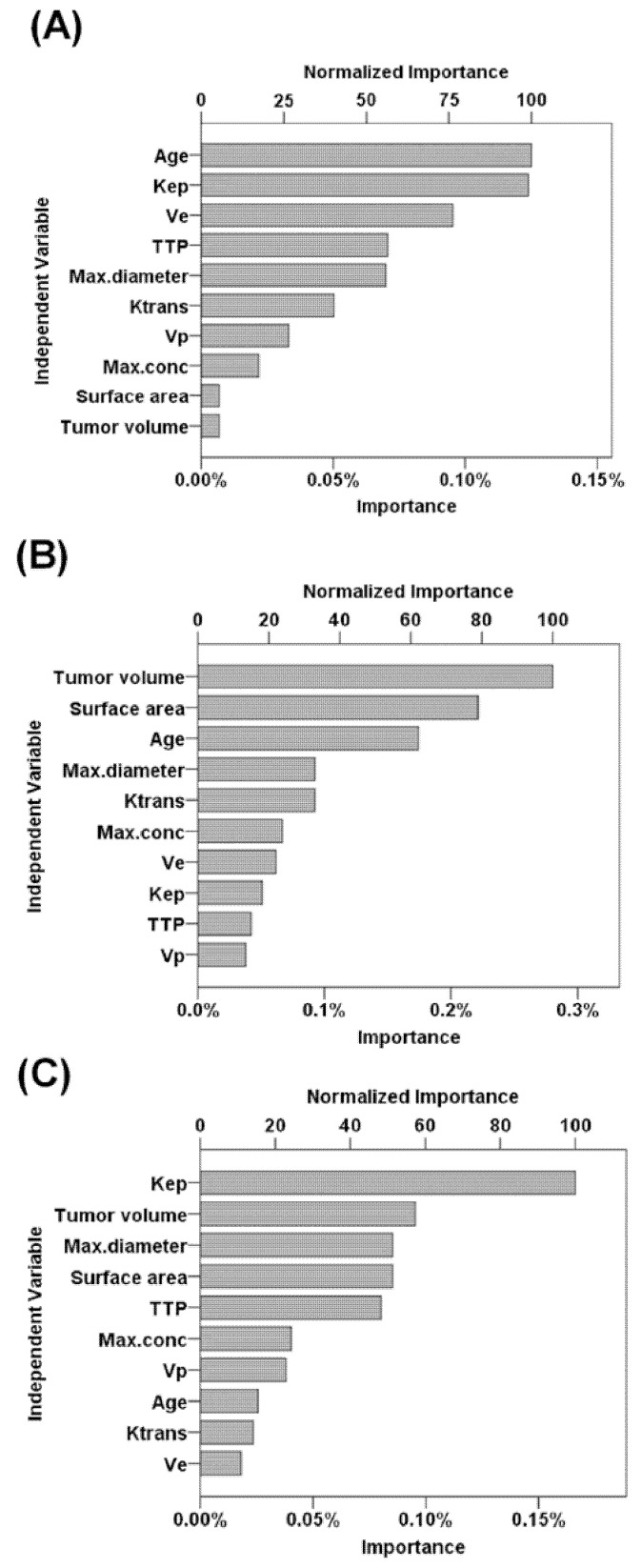
Performance of predictive parameters using variable importance analysis. (**A**) Parameters for predicting PMT subtypes. (**B**) Parameters for predicting Hodgkin lymphoma. (**C**) Parameters for predicting invasive thymoma. Abbreviations: EES = extravascular extracellular space; K_trans_ = efflux rate constant from blood plasma into the tissue EES; K_ep_ = the efflux rate constant from tissue EES into the blood plasma; V_e_= EES volume per unit of tissue; V_p_ = blood plasma volume per unit volume of tissue; TTP = time to peak of the concentration curve.

According to the normalized importance measures, age at MRI examination, K_ep_, and V_e_ were the three most important parameters for predicting PMT subtypes (normalized importance measure = 100%, 99%, and 76.2%, respectively; Figure 2A). In addition, tumor volume and K_ep_ were the most important parameters for predicting Hodgkin lymphoma and invasive thymoma, respectively (both normalized importance measure = 100%; Figure 2B,C).

### 3.5. Predictive Models

**Figure 3:**
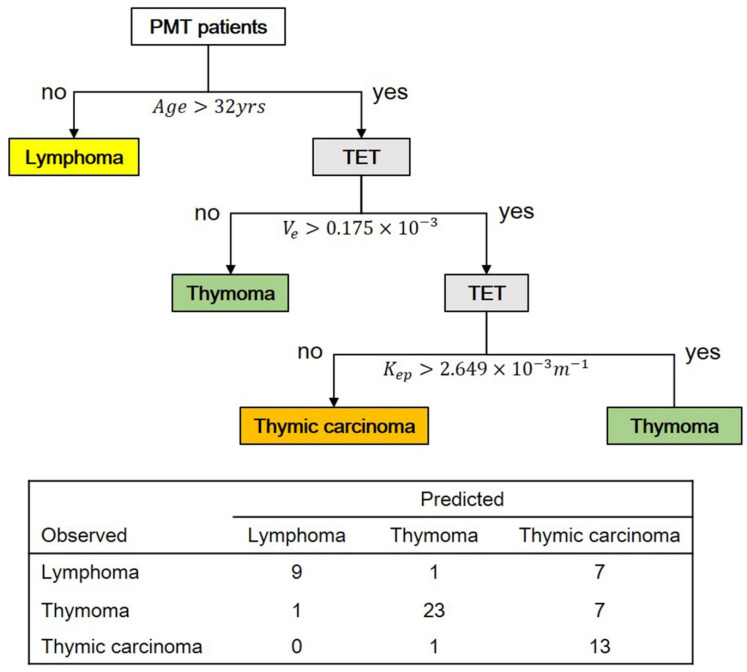
The predictive model for differentiating three PMT subtypes (lymphoma, thymoma, and thymic carcinoma) based on 62 PMT patients. The total predictive accuracy was 72.58%. Abbreviation: TET = thymic epithelial tumor.

Based on multi-class classification, a predictive model for differentiating between PMT subtypes (lymphoma, thymoma, and thymic carcinoma) was constructed using decision tree analysis (Figure 3). Ten parameters including age, MR-derived perfusion parameter, and tumor dimension data were considered while building this decision tree model. Based on a depth setting as level of 3, the first decision node was age with a cut-off of 32 years, the second was V_e_ with a cut-off of 0.175 × 10^−3^ min^−1^, and the third was K_ep_ with a cut-off of 2.649 × 10^−3^ min^−1^. The sensitivities for detecting lymphoma, thymoma, and thymic carcinoma were 52.9%, 74.2%, and 92.8%, respectively. The specificities for detecting lymphoma, thymoma, and thymic carcinoma were 97.8%, 93.5%, and 70.8%, respectively. The total prediction accuracy of this predictive model was 72.58% (Figure 3).

**Figure 4:**
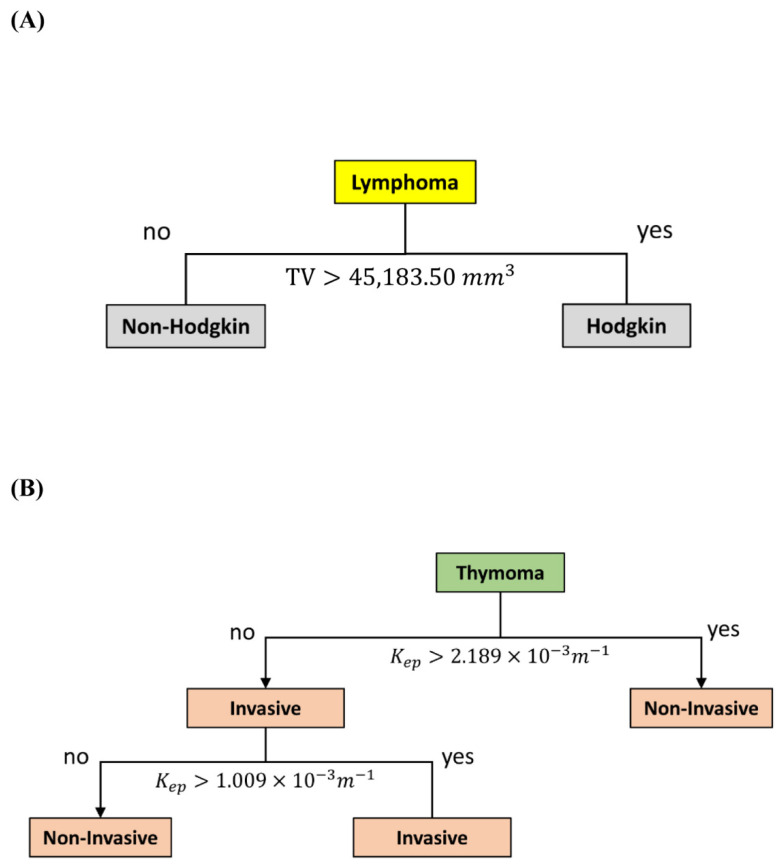
Decision tree models. (**A**) The model for differentiating Hodgkin from non-Hodgkin lymphoma with a total prediction accuracy of 88.24%. (**B**) The model for differentiating invasive from noninvasive thymoma with a total prediction accuracy of 90.32%. Abbreviation: K_ep_ = efflux rate constant from tissue EES into the blood plasma.

In addition, binary classification was applied to build two additional predictive models. Consistent with the finding that tumor volume was the most sensitive parameter in distinguishing between Hodgkin and non-Hodgkin lymphoma with AUC of 0.848 (Supplementary Table S2), the decision tree analysis revealed that the decision node for distinguishing Hodgkin from non-Hodgkin lymphoma was tumor volume with a cut-off of 45183.50 mm^3^, a sensitivity of 75%, a specificity of 100%, and a total prediction accuracy of 88.24% (Figure 4A).

Furthermore, 31 thymoma patients were used to build a decision tree model for distinguishing between patients with invasive and patients with noninvasive forms of thymoma. Based on a depth setting as level of 2, the first decision node was K_ep_ with a cut-off of 2.1489 × 10^−3^ m^−1^, and the second one was K_ep_ with a cut-off of 1.009 × 10^−3^ m^−1^. This predictive model had a sensitivity of 71.4%, a specificity of 95.8%, and a total prediction accuracy of 90.32% (Figure 4B).

### 3.6. Multivariate ROC Curve Analysis

The multivariate ROC curve analysis based on the selected parameters revealed that the predicted performance was 0.864, 0.636, and 0.720 for predicting lymphoma, thymoma, and thymic carcinoma, respectively (Supplementary Table S3). The multinominal logistic regression analysis also indicated that age and K_ep_ were significant risk factors for predicting thymoma and that K_ep_ was a significant risk factor for predicting thymic carcinoma (all *p* value < 0.05, Supplementary Table S4).

## 4. Discussion

In the present retrospective pilot study, 10 parameters, including age, tumor size information, and DCE-MRI–derived perfusion parameters were selected as candidate model parameters; predictive models were then built using a decision tree algorithm. The main predictive model contains 3 levels of split, in which lymphomas, thymoma, and thymic carcinoma are sequentially distinguished based on age, V_e_, and K_ep_, respectively. The sensitivities for detecting lymphoma, thymoma, and thymic carcinoma were 52.9%, 74.2%, and 92.8%, respectively, and the total prediction accuracy was 72.58%. The importance of age, V_e_, and K_ep_ in modelling was confirmed by polytomous logistic regression analysis and variable importance analysis. In addition, two relatively simple predictive models were also built. The first model is able to differentiate between Hodgkin lymphoma and non-Hodgkin lymphoma based on tumor volume, with a sensitivity of 75% and a total prediction accuracy of 88.24%. The second model is capable of distinguishing invasive thymoma from noninvasive thymoma based on K_ep_, with a sensitivity of 71.4% and a total prediction accuracy of 90.32%.

Definitive diagnosis of PMT subtypes is critical for physicians and patients so that they can select the most suitable treatment(s) [13,14,36]. National Comprehensive Cancer Network (NCCN) recommended that resectable thymic tumors should be treated with complete resection, because potential tumor seeding may occur if the tumor capsule is violated during biopsy [26]. Medical imaging modalities may facilitate preoperative differential diagnosis of PMT subtypes [3,33]. Notably, DCE-MRI has been particularly useful for assessing vascular status via perfusion parameters [19,20]. Correlations between DCE-MRI–derived perfusion parameters and histopathological characteristics have been demonstrated in breast cancer [37,38]. Moreover, DCE-MRI–derived perfusion parameters were suggested as imaging biomarkers of angiogenesis prognosis in breast cancer, lung cancer, and rectal cancer [37,38,39,40].

A retrospective study evaluating the differential diagnostic value of DCE-MRI–derived perfusion parameters found that patients with thymic carcinoma had significantly lower K_ep_ and higher V_e_ compared to those with lymphoma; the combination of K_ep_ and V_e_ significantly improved the diagnostic performance, resulting in a sensitivity of 57.1% and a specificity of 93.3% [24]. A recent study reported that among perfusion parameters, K_trans_ had the highest diagnostic accuracy at 74.2%, and a sensitivity of 65.2% in predicting malignancy in solid pulmonary lesions [15]. In the present study, K_trans_, K_ep_, V_e_, and TTP are significantly different between thymic carcinoma and thymoma. Furthermore, our results indicated that K_ep_ and V_e_ were the best predictors for differentiating PMT subtypes and that K_ep_ was the best predictor for distinguishing between invasive and noninvasive thymoma. Therefore, supporting the findings in other medical conditions [21,22,23], the predictive potential of DCE-MRI–derived perfusion parameters for differential diagnosis was demonstrated again by the present study.

A retrospective study of 409 patients with mediastinal lesions concluded that their clinical presentation and histopathological results were affected by age [34]. Hence, we proposed that age may be a predictor for differential diagnosis of PMT subtypes. In this study, we found that patients with lymphoma were significantly younger that those with TET and that age is a good predictor for differentiating lymphoma from TET. On the other hand, a retrospective study reported that the maximal tumor diameter was significantly larger in lymphoma than in TET and that the maximal tumor diameter was a good predictor for differentiating lymphoma from prevascular mediastinal solid tumors [34]. In addition, the contours and shape of TET were suggested to be predictors of postoperative recurrence and metastasis [41]. Therefore, in addition to maximal tumor diameter, tumor volume and surface area were also considered while building the prediction models in this study. Our predictive model revealed that tumor volume is a good predictor for differentiating between Hodgkin and non-Hodgkin lymphoma.

Machine learning algorithms have been applied to build CT imaging–based predictive models for distinguishing low-risk from high-risk thymoma [42] and differentiating subtypes of TET [43]. Hu et al. [43] found that the sensitivity of models constructed by various machine learning algorithms, including SVM and RF, varied, ranging 0.47% to 0.75%; however, to obtain more self-explanatory and visualizable results [31], white-box decision tree algorithms were applied in this study. Machine learning techniques have rarely been applied in construction of MRI imaging–based predictive models for diagnosis of PMTs. To the best of our knowledge, this study is the first to systematically evaluate predictive values of DCE-MRI–derived perfusion parameters in differentiating three major PMT subtypes using a machine learning approach. Shen et al. (2020) previously reported that K_ep_ and V_e_ were significantly different between thymic carcinoma and lymphoma, but they did not include patients with thymoma in their study [24]. Compared to the above study [24], we evaluated the predictive potential of more perfusion parameters (K_trans_, K_ep_, V_p_, V_e_, TTP, and maximum concentration) while modelling, and we also included thymoma in this study to create a more comprehensive predictive model for differential diagnosis of all three PMT subtypes.

This study has several limitations. First of all, it was a retrospective single institution pilot study with a small sample size. Because PMTs account for less than 1% of tumors [2], it is difficult to include a large number of patients from a single institute. In addition, racial influence on the accuracy of our predictive model cannot be explored in this single-institution study conducted in Taiwan; studies conducted in other geographic areas are needed for comparison. Furthermore, due to the small sample size, the possibility of overfitting while modeling cannot be excluded, and it is impossible to merge three predictive models into one with a great maximum tree depth. Therefore, larger-scale multicenter studies are warranted to confirm the current findings and to build a predictive model for accurately differentiating as many PMT subtypes as possible to improve clinical decision making. Furthermore, performance of models built by various machine learning algorithms have to be compared in order to select the optimal model for differentiating PMT subtypes. Another future direction is to integrate more parameters from other MRI sequences, such as apparent diffusion coefficient value, T1 mapping, and extracellular volume, to enhance the sensitivity and accuracy of the machine leaning–based prediction model for differential diagnosis of various PMT subtypes.

## 5. Conclusions

This study systematically evaluates predictive values of DCE-MRI–derived perfusion parameters in differentiating pathological subtypes of PMTs using a machine learning approach. In addition to two perfusion parameters (V_e_ and K_ep_), age and tumor volume were important predictors. The predictive model for differentiating three major PMT subtypes had a 52.9% sensitivity to detect lymphoma, a 74.2% sensitivity to detect thymoma, and a 92.8% sensitivity to detect thymic carcinoma. The total accuracy rate of this predictive model was 72.58%. The results of this pilot study demonstrated the feasibility of machine learning–based predictive models for distinguishing PMT subtypes, which might provide insights into the development of artificial intelligence–based clinical decision support systems for differential diagnosis of distinct PMT subtypes.

## Figures and Tables

**Table 1:** Demographic and clinical characteristics of 62 patients with PMTs.

Variables	Number (%)
Sex	
Male	28 (45)
Female	34 (55)
Age (yr)	52.3 ± 15.8 (22 to 82)
Treatment	
Surgery	34 (54.8)
Chemotherapy	28 (45.2)
PMT subtype	
Lymphoma	17 (27.4)
TET	45 (72.6)
Lymphoma subtype ^a^	
Hodgkin	6 (35.3)
Non-Hodgkin	11 (64.7)
TET subtype ^b^	
Thymoma	31 (68.9)
Thymic carcinoma	14 (31.1)
Invasiveness of thymoma ^c^	
Noninvasive	25 (80.6)
Invasive	6 (19.4)

Data are presented as mean ± standard deviations (range: min. to max.) for age, and n (%) for others. Abbreviations: PMT: prevascular mediastinal tumor; yr: year; TET: thymic epithelial tumor. ^a^ included 17 patients with lymphoma only. ^b^ included 45 patients with TET only. ^c^ included 31 patients with thymoma only.

**Table 2:** Comparisons of age, MR-derived perfusion parameters, and tumor size data between patients with lymphoma and TET and between thymoma and thymic carcinoma.

Variable	Lymphoma (*n* = 17)	TET (*n* = 45)	*p* Value	Thymoma (*n* = 31)	Thymic Carcinoma (*n* = 14)	*p* Value
Age (yr)	30 (26, 48)	59 (52, 65)	<0.001 *^†^	56 (49, 65)	62 (55, 69)	0.169
K_trans_ (10^−3^ min^−1^)	0.34 (0.11, 1.13)	0.46 (0.22, 0.62)	0.664	0.36 (0.17, 0.58)	0.51 (0.45, 1.50)	0.042 *
K_ep_ (10^−3^ min^−1^)	0.86 (0.67, 1.73)	1.70 (0.90, 2.96)	0.073	2.72 (1.14, 4.71)	0.93 (0.72, 1.38)	0.005 *^†^
V_p_ (10^−3^)	0.01 (0.01, 0.03)	0.02 (0.01, 0.05)	0.444	0.02 (0.01, 0.05)	0.03 (0.02, 0.07)	0.086
V_e_ (10^−3^)	0.39 (0.13, 1.01)	0.20 (0.08, 0.54)	0.253	0.13 (0.06, 0.31)	0.52 (0.20, 2.36)	0.001 *^†^
TTP (× 10^2^ s)	1.29 (1.05, 1.96)	1.09 (0.76, 1.75)	0.087	0.89 (0.66, 1.29)	1.72 (1.01, 1.96)	0.003 *^†^
Max. conc. (10^−3^ mM)	32 (18, 47)	21 (11, 38)	0.246	17 (9, 33)	31 (16, 70)	0.062
Tumor volume (× 10^4^ mm^3^)	4.50 (2.06, 6.37)	1.21 (0.57, 4.52)	0.028 *	1.10 (0.49, 4.40)	1.60 (0.67, 5.06)	0.624
Surface area (× 10^4^ mm^2^)	2.49 (1.55, 3.84)	0.80 (0.44, 2.78)	0.027 *	0.72 (0.42, 2.86)	1.14 (0.55, 2.80)	0.573
Max. diameter (× 10^2^ mm)	0.76 (0.65, 1.02)	0.45 (0.35, 0.71)	0.001 *^†^	0.43 (0.35, 0.72)	0.51 (0.41, 0.72)	0.315

Data are presented as median (inter-quartiles) and compared between two groups using the Mann–Whitney U test. Key: K_ep_ = efflux rate constant from tissue EES into the blood plasma; K_trans_ = efflux rate constant from blood plasma into the tissue EES; V_p_ = blood plasma volume per unit volume of tissue; V_e_ = EEs volume per unit volume of tissue; TTP = time to the peak of the concentration curve; TET = thymic epithelial tumor. * *p* < 0.05. ^†^ indicated significant difference after controlling the false discovery rate.

## Data Availability

The data that support the findings of this study are available on request from the corresponding author.

## Supplementary Materials

The following supporting information is available online at can be download at: https://www.mdpi.com/article/10.3390/diagnostics12040889/s1, Table S1: Summary of decision tree hyper-parameters; Table S2: Summary of univariate ROC curve analysis results for distinguishing between PMT subtypes; Table S3: Results of multivariate ROC curve analysis for the classification of PMT subtypes; Table S4: Polytomous logistic regression among all 62 patients to evaluate PMT subtypes with selected parameters derived from the decision tree.

## Abbreviations

AUC	area under the curve
CRAT	classification and regression tree
DCE-MRI	dynamic contrast-enhanced magnetic resonance imaging
EES	extravascular extracellular space
K_ep_	efflux rate constant from tissue EES into the blood plasma
K_trans_	efflux rate constant from blood plasma into the tissue EES
PMT	prevascular mediastinal tumor
RF	random forest
ROC	receiver-operating-characteristic
ROI	region of interest
SVM	support vector machine
TET	thymic epithelial tumor
TTP	time to the peak of the concentration curve
V_e_	EES volume per unit volume of tissue
V_p_	blood plasma volume per unit volume of tissue
