# Supplementary material for: An MRI-Based Clinical-Perfusion Model Predicts Pathological Subtypes of Prevascular Mediastinal Tumors
Source: Diagnostics (Basel). 2022 Apr 2;12(4):889. doi: 10.3390/diagnostics12040889 (PMC9026802; doi:10.3390/diagnostics12040889)
Supplement: Supplementary file 1 [file diagnostics-12-00889-s001.zip › diagnostics-1607962-supplementary.pdf]

Supplementary Table S1. Summary of decision tree hyper-parameters

Hyper-parameters	Model I (Figure 3)	Model II (Figure 4A)	Model III (Figure 4B)
Dependent variable	PMT subtypes	Hodgkin lymphoma	Invasive thymoma
Growing method	CART	CART	CART
Validation	10 fold cross-validation	10 fold cross-validation	10 fold cross-validation
Maximum tree depth	3	1	2
Minimum cases in parent node	7	3	3
Minimum cases in child node	3	1	1
Gini impurity	0.0001.	0.0001.	0.0001.

Abbreviation: CART, Classification and Regression Tree

Supplementary Table S2. Summary of ROC curve analysis results for distinguishing between PMT subtypes*

Parameters	TET vs. lymphoma	Thymic carcinoma vs. thymoma	Hodgkin vs. non-Hodgkin lymphoma	Invasive vs. noninvasive thymoma
Age	0.832 (0.696, 0.968)	0.629 (0.459, 0.799)	0.811 (0.606, 1)	0.623 (0.429, 0.818)
K_{trans}	0.536 (0.365, 0.707)	0.691 (0.532, 0.851)	0.636 (0.346, 0.927)	0.580 (0.323, 0.837)
K_{ep}	0.648 (0.492, 0.804)	0.765 (0.626, 0.904)	0.667 (0.397, 0.936)	0.773 (0.615, 0.932)
V_p	0.563 (0.399, 0.727)	0.661 (0.503, 0.819)	0.515 (0.224, 0.806)	0.533 (0.286, 0.781)
V_e	0.595 (0.431, 0.759)	0.802 (0.663, 0.941)	0.561 (0.280, 0.842)	0.573 (0.325, 0.821)
TTP	0.642 (0.493, 0.792)	0.779 (0.639, 0.918)	0.591 (0.292, 0.890)	0.813 (0.664, 0.962)
Max. conc.	0.596 (0.441, 0.751)	0.675 (0.508, 0.842)	0.652 (0.383, 0.920)	0.513 (0.286, 0.741)
Tumor volume	0.682 (0.547, 0.818)	0.546 (0.369, 0.723)	0.848 (0.648, 1)	0.793 (0.619, 0.968)
Surface area	0.684 (0.549, 0.818)	0.553 (0.377, 0.729)	0.833 (0.630, 1)	0.800 (0.639, 0.961)
Max. diameter	0.780 (0.648, 0.913)	0.594 (0.421, 0.768)	0.788 (0.528, 1)	0.820 (0.660, 0.980)

*Results are presented as AUC (95% confidence intervals).

Supplementary Table S3. Results of multivariate ROC curve analysis for the classification of PMT subtypes

Classification	AUC (95% confidence intervals)
Lymphoma vs. non-lymphoma	0.864 (0.736, 0.992)
Thymoma vs. non-thymoma	0.636 (0.492, 0.779)
Thymic carcinoma vs. non-thymic carcinoma	0.720 (0.582, 0.859)

Multivariate ROC curve analysis was performed based on the selected parameters, including age at diagnosis, V_e, and K_{ep}.

Supplementary Table S4. Polytomous logistic regression among all 62 patients to evaluate PMT subtypes with selected parameters derived from the decision tree

Parameters [a]	Thymoma [R1]	Thymic carcinoma [R1]	Thymic carcinoma [R2]
Age (vs. ≤32)	0.014 (0.001, 0.245)**	NA	NA
V_e (vs. $\leq 0.175 \times 10^{-3}$)	0.383 (0.051, 2.849)	NA	NA
K_{ep} (vs. $\leq 2.649 \times 10^{-3}$)	9.751 (1.008, 94.367)*	0.907 (0.045, 18.444)	0.093 (0.010, 0.902)*

Results are presented as odd ratio with 95% confidence interval (95% confidence intervals) via multinominal logistic regression analysis.

[a] Age at MRI scan, V_e, and K_{ep} were stratified according to the cutoff of 32 years, 0.175×10^{-3}, and 2.649×10^{-3} min^{-1}, respectively, which were used in the classification model shown in Figure 1.

[R1] = reference group: lymphoma, as a dependent variable.

[R2] = reference group: thymoma, as a dependent variable.

NA, not available.

*P < 0.05; **P < 0.05, indicated significant difference.
